# Targeting glioblastoma mitochondrial metabolism with S-Gboxin induces cytotoxicity under conditions of the tumor microenvironment

**DOI:** 10.1038/s41420-026-03072-4

**Published:** 2026-03-27

**Authors:** Jan-Béla Weinem, Hans Urban, Benedikt Sauer, Tanja Buhlmann, Ann-Christin Hau, Stefan Liebner, Tillmann Rusch, Dmitry Namgaladze, Leander F. Harwart, Jan-Hendrik Schröder, Maeve de Souza, Joachim P. Steinbach, Stefan Legewie, Anna-Luisa Luger, Michael W. Ronellenfitsch

**Affiliations:** 1 Dr. Senckenberg Institute of Neurooncology, University Hospital, Goethe University Frankfurt, Frankfurt am Main, Germany; 2 University Cancer Center Frankfurt (UCT), University Hospital, Goethe University Frankfurt, Frankfurt am Main, Germany; 3 German Cancer Consortium (DKTK), Partner Site Frankfurt/Mainz, Frankfurt am Main, Germany; 4 Frankfurt Cancer Institute (FCI), University Hospital, Goethe University Frankfurt, Frankfurt am Main, Germany; 5 Department of Systems Biology, Institute for Biomedical Genetics (IMBG), University of Stuttgart, Stuttgart, Germany; 6 Edinger Institute (Institute of Neurology), University Hospital, Goethe University Frankfurt, Frankfurt am Main, Germany; 7 Department of Hematology, Oncology and Immunology, Philipps University Marburg, Marburg, Germany; 8 Institute of Biochemistry 1, Faculty of Medicine, Goethe University Frankfurt, Frankfurt am Main, Germany

**Keywords:** Cancer metabolism, Targeted therapies, Cancer microenvironment, CNS cancer

## Abstract

Glioblastoma (GB) is the most common primary malignant brain tumor in adults. Gboxin, a novel compound that targets oxidative phosphorylation via complex V inhibition, has shown promise in preclinical models of GB. We examined the efficacy of the pharmacokinetically optimized S-Gboxin under conditions replicating the GB microenvironment, including nutrient deprivation and hypoxia. We assessed cytotoxicity and growth-inhibitory effects of S-Gboxin in human GB cell lines, primary GB cultures, as well as immortalized and primary human astrocytes under different nutrient and oxygen deprivation scenarios. Oxygen consumption, cell migration, activation of the integrated stress response (ISR) as well as the relevance of the AMP-activated protein kinase (AMPK) were evaluated as variables under S-Gboxin treatment. S-Gboxin demonstrated cytotoxicity at low micromolar concentrations, with cell death enhanced under nutrient deprivation and hypoxia. S-Gboxin reduced cellular oxygen consumption and uncoupled mitochondria. Cytotoxicity was increased when mitochondrial fuels were the primary energy source. Additionally, S-Gboxin treatment resulted in elevated lactate production and glucose consumption. While the ISR marker ATF4 was induced by S-Gboxin in a dose-dependent manner, ISR inhibition with ISRIB did not affect its cytotoxicity. Conversely, S-Gboxin treatment combined with AMPK inhibition resulted in enhanced tumor cell death. Collectively, these findings demonstrate that S-Gboxin selectively targets cancer-specific metabolic vulnerabilities in GB cells. The synergistic action with AMPK inhibition suggests that this pathway contributes to maintain energy homeostasis in the presence of the drug. Therefore, S-Gboxin is a promising compound for GB therapy, especially in a combinatory approach with AMPK inhibition or other metabolic targeted therapies.

## Introduction

Glioblastoma (GB) is the most common primary malignant brain tumor in adults, with a dismal prognosis despite multimodal treatment approaches and an evolving clinical trial landscape [1]. Standard treatment typically includes surgical resection followed by radiotherapy combined with temozolomide chemotherapy, yielding median survival times below 15 months [2] and a 5-year survival rate of only 5% [3, 4]. Therapy efficacy is frequently limited by intrinsic and acquired resistance as well as by off-target toxicity. Therefore, there is a great need for new treatment approaches with improved tumor specificity and a favorable side effect profile. Reprogramming of metabolism has recently been recognized as a hallmark of cancer [5], and targeting metabolism by exploiting fundamental differences between tumor cells and non-tumor cells [6] emerged as a promising strategy in cancer research. Glucose is a primary cellular energy source whose metabolism via glycolysis and subsequently the citric acid cycle and oxidative phosphorylation in mitochondria produces ATP. The notion that in tumor cells, glucose is metabolized only via glycolysis with pyruvate/lactate as the end product regardless of oxygen availability, known as the Warburg effect, has been challenged in GB [7]. In the GB tumor microenvironment, where glucose and nutrients are frequently scarce, an efficient utilization of glucose—including the citric acid cycle and oxidative phosphorylation in mitochondria—may be essential for tumor cell survival and proliferation.

Gboxin is a novel compound that was identified in a large-scale drug screen employing a preclinical murine model for its specificity to inhibit oxidative phosphorylation in GB cells while sparing non-tumor cells [8]. Effects were also validated in a panel of human cell lines [8]. Mechanistically, Gboxin targets complex V (ATP synthase) of the mitochondrial respiratory chain, inhibiting ATP production. This inhibition shifts energy dependence in GB cells towards anaerobic pathways, such as glycolysis [8–10]. The tumor cell specificity of Gboxin appears to rely on a non-functional mitochondrial permeability transition pore (mPTP) in GB and other cancer cells [8, 11]. Consequentially, this triggers an increased mitochondrial membrane potential which induces enrichment of Gboxin inside the mitochondria to inhibit respiration and ultimately trigger cell death [8].

Following the discovery of Gboxin, a pharmacokinetically optimized analog, S-Gboxin, was developed for in vivo applications. In a murine GB model, S-Gboxin limited tumor growth and extended survival [8]. However, the effects of both Gboxin and S-Gboxin have not been studied under the nutrient-deprived and severely hypoxic conditions characteristic for the GB tumor microenvironment.

Treatment resistance is a general problem in GB and targeted therapy approaches can induce adaptive mechanisms to limit their efficacy or even parallel unwanted tumor cell protective effects [12]. The integrated stress response (ISR) is a critical cellular mechanism that senses nutrient deprivation, hypoxia, and other stressors [13, 14]. Activation of the ISR includes phosphorylation of eIF2α by specific kinases, leading to a global reduction in protein synthesis while simultaneously inducing translation of specific mRNAs including the transcription factor ATF4 as the master regulator of the ISR [13]. ATF4 interacts via its leucine zipper domain to activate genetic adaptation programs, which can include target genes with roles in metastasis, amino acid synthesis, and angiogenesis [14]. In prior studies in murine cells and different human cell lines, Gboxin was shown to induce *ATF4* expression, suggesting its activation of the ISR pathway [8]. AMP-activated protein kinase (AMPK) is a central cellular energy sensor activated by a decline in ATP levels [15, 16]. AMPK promotes preserving energy by reducing expenditure and increasing generation of ATP e.g., by promoting mitochondrial biogenesis via the transcription factor Peroxisome Proliferator-activated Receptor gamma Coactivator 1-alpha (PGC-1α) [15–18] or inhibition of mTOR, a master regulator of translation and cell growth [16, 19]. Previously, Gboxin treatment was shown to activate AMPK in line with its metabolic effects [8].

In this study, we aimed to evaluate the effects of S-Gboxin on human GB cells, hypothesizing that its cytotoxicity is influenced by oxygen availability and nutrient conditions, with reduced cell survival in the absence of mitochondrial substrates. Additionally, we evaluated the potential of a co-treatment approach with inhibition of the ISR or AMPK.

## Results

### S-Gboxin is cytotoxic in human glioblastoma cell lines

**Fig. 1:**
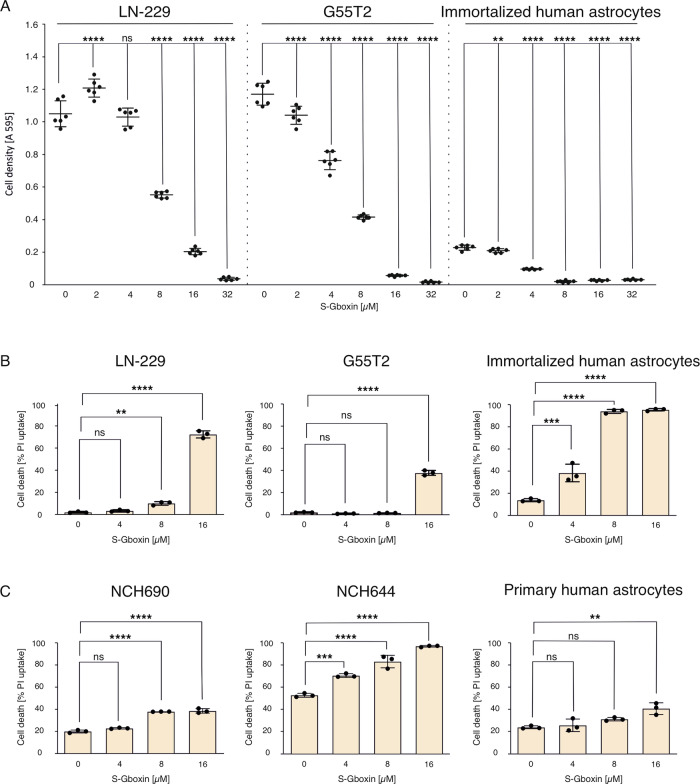
S-Gboxin induces cytotoxicity and cell death in human glioma cell lines and glioblastoma stem-like cells. **A** LN-229 cells, G55T2 cells and immortalized human astrocytes were treated in serum-free medium for 24 h with 2 µM to 32 µM S-Gboxin. Cell density was measured by CV-staining. For control, cells were treated with the equal amount of DMSO compared to the highest S-Gboxin concentration (*n* = 6, Mean and S.D. are presented. ns = not significant, *p*-value *<0.05, **<0.01, ***<0.001, ****<0.0001; one-way ANOVA with Tukey’s multiple comparison). **B** Cell death of LN-229 cells (left panel), G55T2 cells (middle panel) and immortalized human astrocytes (right panel) were analyzed by PI-staining. The cells were treated with 4 µm, 8 µM and 16 µM S-Gboxin for 24 h in serum-free medium. The equal amount of DMSO compared to 16 µM S-Gboxin was used as control (*n* = 3, Mean and S.D. are presented. ns = not significant, *p*-value *<0.05, **<0.01, ***<0.001, ****<0.0001; one-way ANOVA with Tukey’s multiple comparison). **C** Similar, cell death of glioblastoma stem-like cells NCH690 (left panel) and NCH644 (middle panel) was analyzed. NCH644 cells were treated for 26 h with 4 µM, 8 µM and 16 µM S-Gboxin in neurobasal-medium. NCH690 cells were treated under the same S-Gboxin concentrations for 24 h in neurobasal-medium. Primary human astrocytes were treated under the same conditions in astrocyte growth medium (right panel) (*n* = 3, Mean and S.D. are presented. ns = not significant, *p*-value *<0.05, **<0.01, ***<0.001, ****<0.0001; one-way ANOVA with Tukey’s multiple comparison).

To assess the efficacy of S-Gboxin relative to the original Gboxin compound, GB cell lines were incubated with varying concentrations of S-Gboxin (Fig. 1). S-Gboxin demonstrated a strong effect in decreasing cell density (Fig. 1). In LN-229 cells, reduced cell density and increased cytotoxicity were observed at 8 µM after 24 h, with a marked cell density reduction occurring at concentrations of 16 µM or higher (Fig. 1A). Similar responses were noted in G55T2 cells (Fig. 1A). Evaluation of immortalized human astrocytes revealed comparable reductions in cell density at S-Gboxin concentrations between 8 µM and 32 µM (Fig. 1A).

To examine whether S-Gboxin induces cell death in human GB cells, we treated LN-229 and G55T2 cells, immortalized human astrocytes, and GB stem-like cells (NCH690 and NCH644) as well as primary human astrocytes with increasing concentrations and quantified cell death (Fig. 1B, C). S-Gboxin was more potent in LN-229 cells (73% cell death at 16 µM) than in G55T2 cells (38% cell death) (Fig. 1B). In LN-229 cells, 8 µM S-Gboxin increased PI-positive cells to 10% compared to 2% in the control (Fig. 1B). In contrast, G55T2 cells showed no enhanced cell death at 8 µM S-Gboxin (Fig. 1B). Notably, immortalized human astrocytes were also sensitive to S-Gboxin even at a 4 µM concentration with a 25% increase in cell death compared to control (Fig. 1B). Gboxin was less potent than S-Gboxin in human glioma cell lines (Supplementary Fig. 2, Fig. 1B). We next investigated S-Gboxin cytotoxicity in GB stem-like cells (Fig. 1C). Similar to effects in established glioma cell lines, enhanced cell death was detectable under 8 µM S-Gboxin in both NCH690 and NCH644 cultures (Fig. 1C). Notably in NCH644 cells, effects were observed down to 4 µM, with cell death escalating at concentrations up to 16 µM (Fig. 1C). In line with a potential selectivity for immortalized/transformed cells, toxic effects in primary human astrocytes were clearly reduced (Fig. 1C). This selectivity was also detected when cells were incubated in primary human astrocyte growth medium (Supplementary Fig. 3A). In summary, S-Gboxin reduced cell density in human GB cell lines and immortalized human astrocytes (Fig. 1A) and induced cell death in both established GB cell lines and GB stem-like cells as well as immortalized human astrocytes whereas effects in primary (non-immortalized) human astrocytes were strongly reduced (Fig. 1B, C).

### Glucose restriction and hypoxia enhance the cytotoxicity of S-Gboxin

**Fig. 2:**
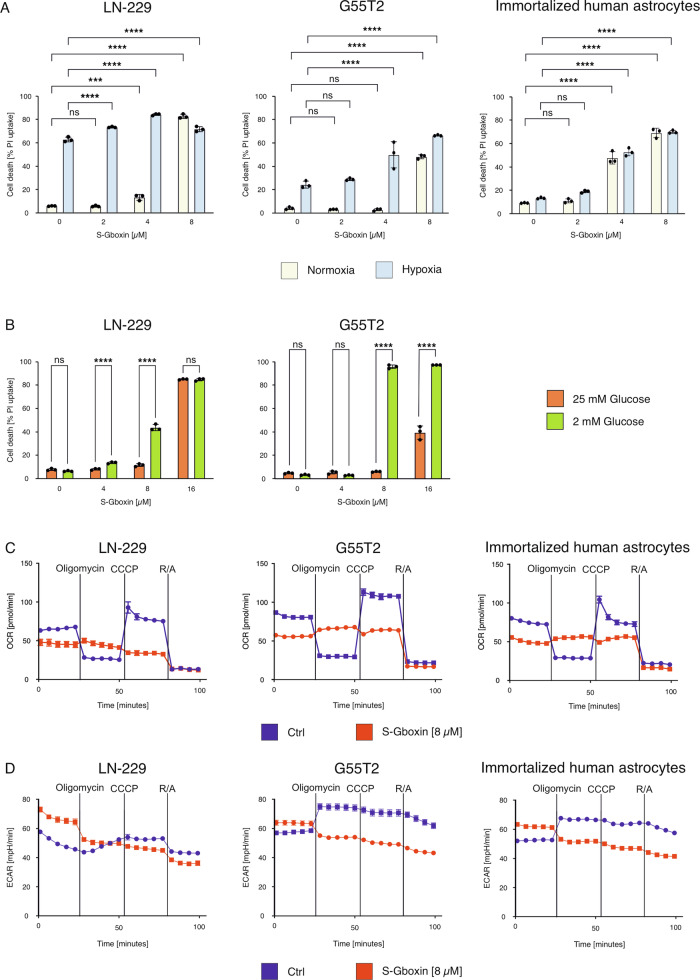
Effects of S-Gboxin are enhanced under the conditions of the tumor microenvironment. **A** LN-229 (left panel), G55T2 (middle panel) and immortalized human astrocytes (right panel) were treated with 2 µM, 4 µM and 8 µM S-Gboxin in serum-free medium containing 2 mM glucose. LN-229 and G55T2 cells were incubated for 20 h under normoxic (21% O_2_, yellow) or hypoxic (0.1% O_2_, blue) conditions. Immortalized human astrocytes were treated under the same conditions for 23 h. Cell death was analyzed by PI staining and flow cytometry (*n* = 3, Mean and S.D. are presented. ns = not significant, *p*-value *<0.05, **<0.01, ***<0.001, ****<0.0001; two-way ANOVA with Tukey’s multiple comparison). **B** LN-229 (left panel) and G55T2 (right panel) were incubated for 24 h in serum-free medium containing 25 mM (orange) or 2 mM glucose (green). Concentration of 4 µM, 8 µM and 16 µM S-Gboxin were used (*n* = 3, Mean and S.D. are presented. ns = not significant, *p*-value *<0.05, **<0.01, ***<0.001, ****<0.0001; two-way ANOVA with Tukey’s multiple comparison). **C** Oxygen consumption rate was analyzed in LN-229 (left panel), G55T2 (middle panel), and immortalized human astrocytes (right panel) pre-treated with 8 µM S-Gboxin and following injections of oligomycin, CCCP, and rotenone/antimycin. **D** Extracellular acidification rate was determined in human glioma cell lines LN-229 (left panel) and G55T2 (middle panel) as well as in immortalized human astrocytes (right panel) after pre-treatment with 8 µM S-Gboxin and following injections of oligomycin, CCCP, and rotenone/antimycin.

To mimic conditions of the tumor microenvironment of GB, we tested the effects of S-Gboxin under glucose restriction and hypoxia (Fig. 2A, B). In LN-229 cells only a minor increase in cell death under glucose restriction and 4 µM S-Gboxin in normoxia was detectable. Treatment with 8 µM however caused a strong increase in cell death above 80% (Fig. 2A). During hypoxia lower S-Gboxin concentrations (2 and 4 µM) were already cytotoxic under glucose-restricted conditions compared to normoxia in LN-229 as well as G55T2 (Fig. 2A) but not in human immortalized astrocytes (Fig. 2A). Treatment with 4 µM S-Gboxin under glucose restriction alone without hypoxia led to elevated cell death in LN-229 cells compared to non-glucose restricted conditions (Fig. 2B). 8 µM S-Gboxin did not induce cell death under glucose-restriction in primary human astrocytes (Supplementary Fig. 3B). In contrast, treatment of G55T2 cells with 8 µM S-Gboxin under glucose restriction enhanced the cell death (Fig. 2B). Glucose restriction also enhanced induction of cell death in the mouse GB-cell line GL-261, which was abolished under glucose-replete conditions (Supplementary Fig. 4).

Based on the results above as well as previously published data [8] we hypothesized that S-Gboxin impaired oxygen-dependent metabolism in human GB cell lines.

We analyzed oxygen consumption rates (OCR) in G55T2, LN-229, and immortalized human astrocyte cell lines following 5 h exposure to 8 µM S-Gboxin using Seahorse-based extracellular flux analysis. Unexpectedly, and in contrast to proposed mechanism of action of Gboxin as ATP synthase inhibitor, OCR profiles of S-Gboxin-pre-treated cells suggested its action as mitochondrial uncoupler (Fig. 2C). Whereas basal respiration was only moderately reduced, cells were insensitive to the ATP synthase inhibitor oligomycin as well as to the mitochondrial uncoupler CCCP. This resulted in a complete loss of ATP-coupled respiration.

S-Gboxin pre-treated cells also showed increased basal rates of extracellular acidification (ECAR) in Seahorse analyses (Fig. 2D). Since basal OCR was attenuated, this indicates increased glycolysis. Interestingly, upon oligomycin injection, ECAR was reduced. This suggests that after S-Gboxin pre-treatment and mitochondrial uncoupling, ATP synthase starts operating in the reverse mode to preserve the mitochondrial potential, thus consuming ATP instead of producing it. The increased ATP demand through reverse operation of ATP synthase is covered by increased glycolysis, thus, explaining ECAR decrease after ATP synthase inhibition in S-Gboxin pre-treated cells. A decreased oxygen consumption was also observed in the fluorescence-based assay (Supplementary Fig. 5).

S-Gboxin-mediated cell death was enhanced under glucose restriction and hypoxia (Fig. 2A, B) demonstrating that S-Gboxin retained its efficacy under conditions of the tumor microenvironment. Adverse effects under such conditions are a problem that is sometimes observed with targeted therapies [20, 21].

### Dependency on mitochondrial energy production enhances S-Gboxin efficacy

**Fig. 3:**
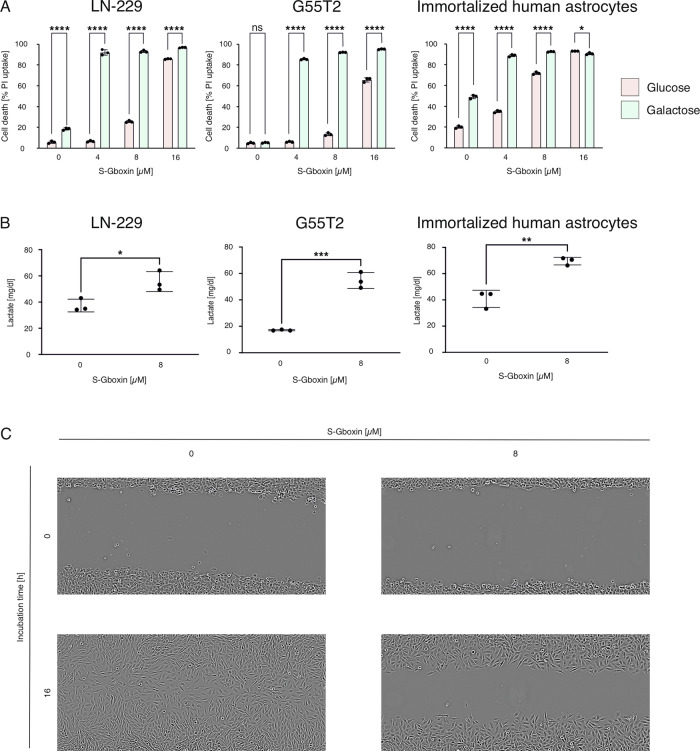
S-Gboxin leads to enhanced cell death with mitochondrial fuels and to a reduced ability of migration. **A** The effects of S-Gboxin under mitochondrial fuels were checked by using 25 mM galactose or 25 mM glucose in LN-229 (left panel), G55T2 (middle panel) or immortalized human astrocytes (right panel). After incubation with 4 µM, 8 µM or 16 µM S-Gboxin in serum-free medium containing either 25 mM glucose (red) or 25 mM galactose (green) for 24 h, cell death was measured by PI stain and flow cytometry (*n* = 3, Mean and S.D. are presented. ns = not significant, *p*-value *<0.05, **<0.01, ***<0.001, ****<0.0001; two-way ANOVA with Tukey’s multiple comparison). **B** Lactate production (displayed in mg/dl) was measured after 10 h treatment with 8 µM S-Gboxin or equal amount of DMSO in serum-free medium containing 25 mM glucose (*n* = 3, Mean and S.D. are presented. p-value *<0.05, **<0.01, ***<0.001, ****<0.0001; student’s *t*-test). **C** The migration of the cells was analyzed after treatment with equal-DMSO amount to 8 µM S-Gboxin or after treatment with 8 µM S-Gboxin. Pictures were taken after 0 h and after 16 h.

Due to the enhancement of S-Gboxin cytotoxicity by low glucose as well as its effects on oxygen consumption we hypothesized that S-Gboxin disables energy production from mitochondrial fuels. We chose galactose whose conversion to glucose is kinetically slow so that cells need to further process galactose via mitochondrial metabolism in order to generate sufficient energy to sustain survival [18]. Using galactose as nutrient source, we observed a strong increase in cell death under 4 µM S-Gboxin in LN-229 (from 18 to 92%) and G55T2 (from 5 to 86%) cells while under glucose replete conditions no such effect was detectable (Fig. 3A). Similar effects were observed with higher concentrations of S-Gboxin (Fig. 3A). Immortalized human astrocytes hardly tolerated galactose as an energy source at all with already 49% dead cells even without S-Gboxin (Fig. 3A). 4 µM S-Gboxin enhanced cell death to 89% (Fig. 3A). In glucose-replete conditions, cell death was enhanced from 20 to 35% (Fig. 3A). 8 µM and 16 µM S-Gboxin enhanced the cell death under glucose (72% and 93%) and galactose (93% and 91%) (Fig. 3A).

### S-Gboxin enhances lactate production and glucose consumption while reducing migration

As a consequence of uncoupled mitochondria and reverse mode of ATP synthase, cells may reduce pyruvate to lactate to restore the NAD+/electron acceptor pool. Additionally, they may increase glucose consumption to compensate for the reduction in ATP when glucose is only metabolized by glycolysis (without the citric acid cycle). Therefore, we measured lactate production and glucose consumption under S-Gboxin. Indeed, 8 µM S-Gboxin enhanced glucose consumption (Supplementary Fig. 6A) and lactate was elevated (Fig. 3B). Notably, cell viability was not affected after shorter periods of S-Gboxin treatment in our tumor cells (Supplementary Fig 6B). Meanwhile, cell migration which is an energy consuming process was reduced by S-Gboxin (Fig. 3C). To exclude confounding effects of cell cycle regulation we performed experiments with S-Gboxin in combination with mitomycin c treatment (Supplementary Fig. 7). At S-Gboxin concentrations of 4 and 8 µM, mitomycin c did not alter the efficacy of S-Gboxin (Supplementary Fig. 7) indicating that cell cycle phase dependency can be considered negligible (Supplementary Fig. 7).

### The integrated stress response does not mediate resistance to S-Gboxin treatment

**Fig. 4:**
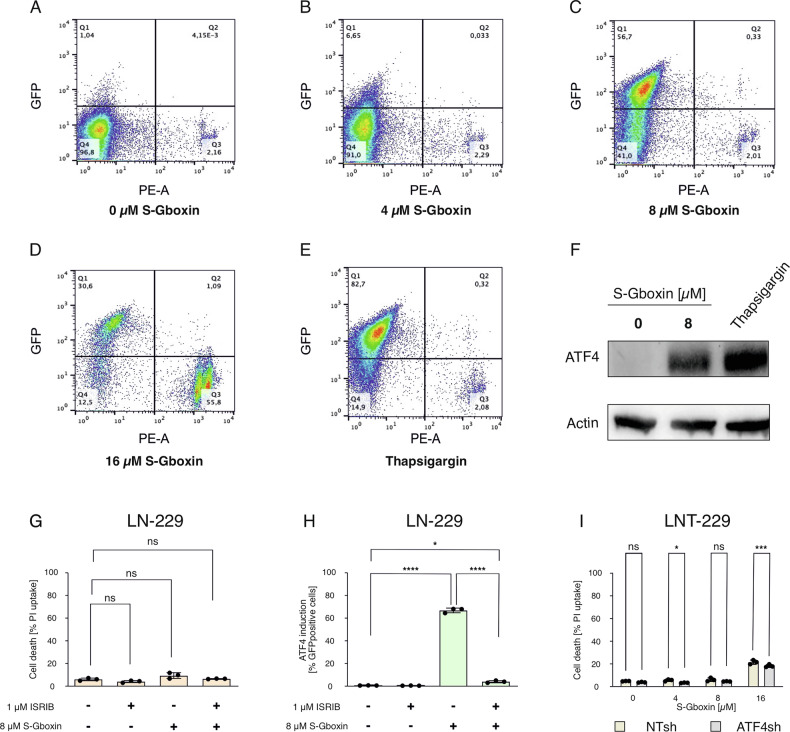
S-Gboxin induces the integrated stress response in human glioma cell lines. **A**–**E** The effect of S-Gboxin on the induction of integrated stress response was measured with GFP-coupled ATF4 LN-229 reporter cells. The cells were treated with 4 µM (**B**), 8 µM (**C**) or 16 µM S-Gboxin (**D**) for 24 h in serum-free medium. Equal amount of DMSO to 16 µM S-Gboxin was used as negative control (**A**) and 1 µM thapsigargin was used as positive control (**E**). The ATF4-induction was measured by flow cytometry (y-axis, GFP) together with PI-stain to detect cell death (x-axis, PE-A). **F** ATF4 induction was also analyzed by immunoblot after 24 h incubation of LN-229 cells in serum-free media with 8 µM S-Gboxin or 1 µM thapsigargin. To determine the effects on cell death with pharmacological inhibition of ATF4, the LN-229 ATF4 reporter cells were treated for 24 h with 8 µM S-Gboxin and/or 1 µM ISRIB (**G**, **H**). Cell death was measured by PI stain and flow cytometry (**G**), ATF4 induction was measured by GFP-signal in flow cytometry (**H**) (*n* = 3, Mean and S.D. are presented. ns = not significant, *p*-value *<0.05, **<0.01, ***<0.001, ****<0.0001; one-way ANOVA with Tukey’s multiple comparison). **I** Effects in *ATF4* gene suppressed cells was measured by PI stain and flow cytometry. The cells were incubated for 24 h in serum-free medium containing 4 µM, 8 µM or 16 µM S-Gboxin. The ATF4 knockdown cells (gray) are compared with control cells (brown) (*n* = 3, Mean and S.D. are presented. ns = not significant, *p*-value *<0.05, **<0.01, ***<0.001, ****<0.0001; two-way ANOVA with Tukey’s multiple comparison).

The ISR is sensitive to a variety of extrinsic stressors including hypoxia and glucose deprivation [13, 14]. A key marker of the ISR is the transcription factor ATF4 which is induced by the ISR to orchestrate adaptive programs [13]. Previously, effects of Gboxin on the ISR have already been suggested [8]. We wondered whether the ISR was also activated in our GB cells as a consequence of S-Gboxin treatment and whether this was a compensatory mechanism to reduce S-Gboxin toxicity. To this end we generated LN-229 ATF4 reporter cells (Supplementary Fig. 1). When treated with S-Gboxin the reporter cells reliably induced the ISR as detected by an increase in GFP signal (Fig. 4A–E). E.g. under 8 µM S-Gboxin a strong induction of the ISR from 1 to 57% positive reporter cells was detectable (Fig. 4A–C). The fact that 16 µM S-Gboxin treatment triggered a comparably lower amount of positive reporter cells was due to an increase in cell death (Fig. 4D). Similar effects were also observed in G55T2 ISR reporter cells (Supplementary Fig. 8B). Thapsigargin, a paradigmatic activator of the ISR was included as positive control and led to 83% GFP positive cells (Fig. 4E). The induction of ISR, measured by upregulation of ATF4, was observed in immunoblot assays as well (Fig. 4F) and was confirmed by fluorescence microscopy of LN-229 GFP-coupled ATF4 reporter cells (Supplementary Fig. 8A). To determine whether ISR induction mitigates S-Gboxin efficacy we investigated effects of its pharmacological and genetic inhibition. Cells were treated with 8 µM S-Gboxin alongside 1 µM ISRIB, an inhibitor of ATF4 activation (Fig. 4G, H). Although ISRIB efficiently inhibited S-Gboxin-induced ATF4 activation (Fig. 4H), it did not increase cell death (Fig. 4G). Extended incubation times and varying S-Gboxin concentrations also failed to alter cell viability in the presence of ISRIB (Supplementary Fig. 9). Furthermore, no enhanced cell death was detectable in LNT-229 *ATF4* gene-suppressed cells [14] (Fig. 4I) in line with the notion that the ISR does not mediate resistance to S-Gboxin despite ATF4 induction in our cell models.

### S-Gboxin treatment synergizes with AMPK inhibition

**Fig. 5:**
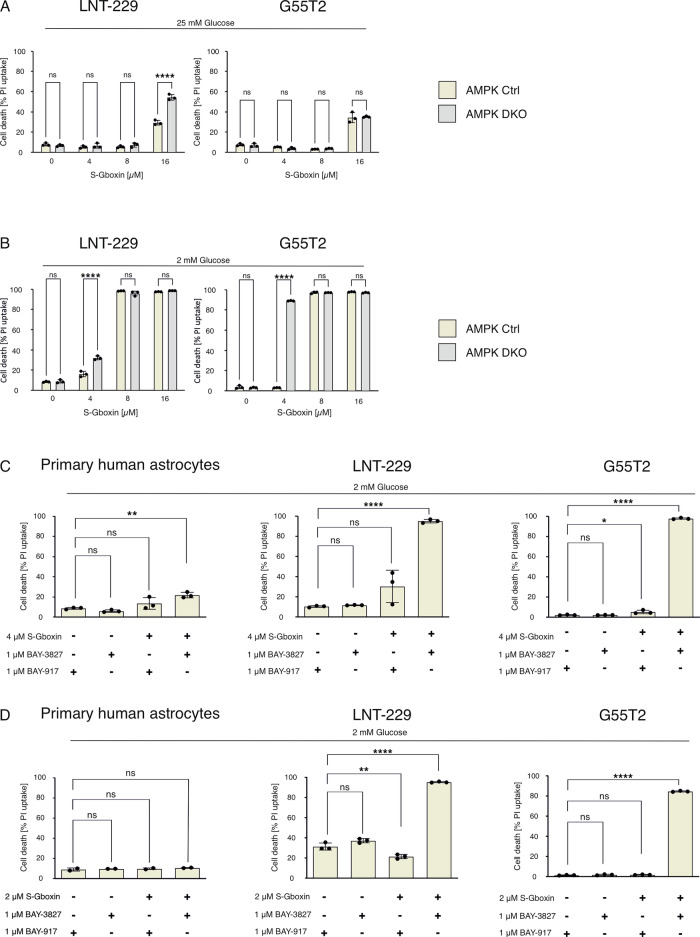
Inhibition of AMPK sensitizes glioblastoma cell lines to S-Gboxin treatment. **A**, **B** Efficacy of S-Gboxin was tested in AMPK-knockout cells. LNT-229 (left panel) or G55T2 (right panel) cells were incubated for 24 h in 25 mM (**A**) or 2 mM glucose containing medium (**B**) in different concentrations of S-Gboxin. Cell death was quantified by PI-staining and FACS-analysis (*n* = 3, Mean and S.D. are presented. ns = not significant, *p*-value *<0.05, **<0.01, ***<0.001, ****<0.0001; two-way ANOVA with Tukey’s multiple comparison). **C** Primary human astrocytes (left panel), LNT-229 cells (middle panel) and G55T2 cells (right panel) were tested under 2 mM glucose and a combinatory pharmacological approach. Cells were treated for 24 h with 4 µM S-Gboxin and/or with 1 µm AMPK-inhibitor BAY-3827 or the control substance BAY-917 (*n* = 3, Mean and S.D. are presented. ns = not significant, *p*-value *<0.05, **<0.01, ***<0.001, ****<0.0001; one-way ANOVA with Tukey’s multiple comparison). **D** The same experiment was repeated with 2 µm S-Gboxin and equal concentrations of BAY-3827 or BAY-917. In both experiments, cell death was quantified using PI and FACS analysis (*n* = 2 or 3, Mean and S.D. are presented. ns = not significant, *p*-value *<0.05, **<0.01, ***<0.001, ****<0.0001; one-way ANOVA with Tukey’s multiple comparison).

AMPK is a central cellular energy sensor that is frequently induced by mitochondrial impairment. Additionally, S-Gboxin has previously been shown to induce AMPK signaling [8]. Therefore, we hypothesized that AMPK inhibition enhanced cell death in response to S-Gboxin. We made use of our previously generated cells with a double knockout of both catalytic (α1 and α2) subunits of AMPK [16]. These knockout cells, similar to the ATF4 knockdown cells mentioned above, were generated from a subclone of LN-229 cells which had retained wildtype TP53 status (LNT-229) [22]. LNT-229 and G55T2 cells were treated with increasing concentrations of S-Gboxin (Fig. 5A, B). When glucose was replete, AMPK knockout enhanced cell death only at the highest concentration of S-Gboxin in LNT-229 cells (Fig. 5A), whereas no notable increase in cytotoxicity was detected in G55T2 cells (Fig. 5A). In contrast, glucose restriction significantly increased cell death in G55T2 cells treated with 4 µM S-Gboxin (Fig. 5B), and similar effects were observed in LNT-229 cells treated with 4 µM S-Gboxin (Fig. 5B) indicating that AMPK-knockout enhances S-Gboxin efficacy under glucose deprivation. Prompted by these results, we examined the impact of pharmacologic AMPK inhibition in GB cells and primary human astrocytes using a novel compound (BAY-3827) with superior specificity and an inactive control (BAY-917) compound [16, 23]. Primary human astrocytes displayed a minimal response to either S-Gboxin or AMPK inhibition alone (Fig. 5C, D). However, combining S-Gboxin with BAY-3827 in GB cell lines LNT-229, G55T2 (Fig. 5D) and LN-229 (Supplementary Fig. 10) produced a dose-dependent, additive cytotoxic effect under 2 mM glucose at 2 µM S-Gboxin. Similar effects are observed under 4 µM S-Gboxin (Fig. 5C). Pharmacological activation of AMPK using A769662 did not lead to a protective effect against S-Gboxin treatment (Supplementary Fig. 11).

## Discussion

GB remains the major challenge in clinical neurooncology. Gboxin is a novel compound that exploits a dysfunctional mPTP to achieve GB cell specificity. Because data on efficacy of this novel compound under realistic conditions of the tumor microenvironment were lacking we evaluated effects under nutrient deprivation and hypoxia as well as dependency on mitochondrial energy sources employing the pharmacokinetically optimized analog S-Gboxin in human GB cells and primary GB cultures.

In our experiments, S-Gboxin effectively reduced cell density in all tested human glioma cell lines (Fig. 1A), with detectable effects in LN-229 and G55T2 cells beginning at concentrations of 4 µM. Interestingly, similar reductions in cell density were also observed in immortalized human astrocytes (Fig. 1A). However, these cells differ from primary (non-transformed) cells by being genetically immortalized as a plausible explanation for their sensitivity to S-Gboxin. Across glioma cell lines, GB stem-like cells and immortalized astrocytes, S-Gboxin was cytotoxic with corresponding effects on cell density (Fig. 1). Notably when we used non-transformed primary human astrocytes instead of immortalized human astrocytes, S-Gboxin was specific for tumor cells and immortalized cells. No cell death was detectable under 4 and 8 µM S-Gboxin (Fig. 1C, last panel). Cell death induced by 16 µm S-Gboxin was drastically higher in glioma cells compared to primary human astrocytes (Fig. 1C). This was also apparent when using astrocyte growth medium (Supplementary Fig. 3A). Moreover, 8 µm S-Gboxin induces a significant higher cell death under glucose starvation in LN-229 cells (Supplementary Fig. 3B). However, in non-transformed primary human astrocytes no induction of cell death under these conditions was detectable (Supplementary Fig. 3B).

A key consideration in exploring new treatment options for GB is their efficacy under the adverse nutrient and oxygen conditions characteristic of the tumor microenvironment. For many targeted therapies, these challenging conditions may undermine efficacy or even promote resistance [12, 20, 21]. At 8 µM, S-Gboxin’s cytotoxicity increased significantly under low-glucose conditions in GB cell lines (Fig. 2A, B), likely due to enhanced cellular dependence on oxidative phosphorylation for ATP generation under glucose restriction. Because S-Gboxin inhibits oxidative phosphorylation, glucose and oxygen depletion led to enhanced cell death, indicating that S-Gboxin may have improved efficacy in the nutrient-depleted tumor microenvironment (Fig. 2A, B).

Furthermore, our results suggest that S-Gboxin uncouples mitochondrial function causing a reversal of ATP synthase activity and thus increasing the cellular dependence on glycolysis for ATP generation (Fig. 2C, D). Our data thus warrants further mechanistic investigation towards understanding the exact molecular targets of S-Gboxin. Also, in line with that, the oxygen consumption was reduced under S-Gboxin (Fig. 2C; Supplementary Fig. 5). In some experimental settings the level of oxygen consumption was positively linked to cell death under hypoxic conditions [24]. Impaired oxygen consumption could therefore lead to reduced cell death under hypoxic conditions. However, despite reducing oxygen consumption (Fig. 2C), S-Gboxin did not confer protection against hypoxia and remained effective in oxygen-depleted environments, further supporting its potential as a targeted agent for GB under typical tumor microenvironment conditions (Fig. 2A–C).

Of note, the experimental setup for Figs. 1 and 2 was optimized to investigate the acute effects of S-Gboxin under nutrient deprivation and hypoxia while maintaining cell viability in the corresponding control conditions requiring a limitation of exposure time. This was a deliberate choice, as the focus of the current manuscript is on acute, mechanistic effects of S-Gboxin and its interaction with metabolic stress pathways. The apparently narrow therapeutic window observed when comparing GB cells with immortalized astrocytes should be interpreted with caution. Immortalization probably alters cellular metabolism and mitochondrial function, rendering these cells a bit more similar to transformed cells. Therefore, we included primary human astrocytes as a non-transformed control.

When glucose is scarce mitochondrial metabolism could ensure maintenance of energy homeostasis. In our experiments we exploited the kinetically slow conversion of galactose to glucose which is a prerequisite for its metabolism. Therefore, galactose indirectly exposes cells to a very low (steady state) glucose level that often necessitates oxidative phosphorylation for sufficient energy [18, 25]. Under mitochondrial metabolism, GB cells were specifically sensitive to S-Gboxin (Fig. 3A) further demonstrating its efficacy under starvation conditions.

In our study, we hypothesized that inhibiting oxidative phosphorylation with S-Gboxin lead to a metabolic shift towards glycolysis and anaerobic pathways, resulting in an accelerated depletion of glucose under low-nutrient conditions and culminating in increased cell death. This metabolic shift was validated by glucose depletion (Supplementary Fig. 6A) and increased lactate levels in the medium (Fig. 3B). These results were corroborated by the uncoupling of mitochondria leading to a reverse mode of action of ATP synthase resulting in increased ATP-dependency to maintain the mitochondrial potential (Fig. 2C, D). Additionally, impaired cell migration (Fig. 3C) was likely due to the energy crisis induced by S-Gboxin. Cell cycle dependency of S-Gboxin efficacy appeared negligible because mitomycin c did not alter S-Gboxin effects on LN-229 and G55T2 cells at 4 or 8 µM (Supplementary Fig. 7).

With regards to cellular resistance mechanism against S-Gboxin treatment, we tested the ISR as well as AMPK signaling as potential mechanisms. Activation of ATF4 in response to S-Gboxin treatment (Fig. 4A–D, F) highlighted an important link to potentially mediate adaptation [14, 26]. However, pharmacological inhibition of ATF4 with ISRIB did not affect cell viability under S-Gboxin treatment (Fig. 4G). Similarly, *ATF4* gene suppression did not enhance S-Gboxin toxicity (Fig. 4I). Therefore, while activation of the ISR takes place under S-Gboxin treatment, it does not protect from S-Gboxin-mediated cell death. In contrast to these results, combination of AMPK inhibition and S-Gboxin was an approach with superior efficacy especially under starvation conditions (Fig. 5). In line, S-Gboxin had no significant effects in lower dose ranges under glucose-replete conditions (Fig. 5A) in contrast to an increased efficacy even at 4 µM S-Gboxin under glucose-restricted conditions in AMPK knockout cells (Fig. 5B). This effect was especially apparent under acute AMPK inhibition with the novel AMPK inhibitor BAY-3827 (Fig. 5C, D). Under acute AMPK inhibition even lower S-Gboxin concentrations were highly effective (Fig.5D). Most likely, cells with chronic AMPK inhibition i.e., AMPK DKO cells, have activated at least some adaptive mechanisms to compensate for the lack of AMPK activity which could explain why pharmacological AMPK inhibition is even more effective in sensitizing cells to S-Gboxin. Nevertheless, both pharmacological as well as genetic AMPK inhibition did sensitize tumor cells to S-Gboxin. Remarkably, no sensitization by AMPK inhibition could be detected in primary human astrocytes indicating a tumor-cell specificity of this approach (Fig. 5C, D).

Gboxin also possesses several pharmacokinetic disadvantages including its short halftime in the body [27].Therefore S-Gboxin was developed [8]. Another limiting factor for Gboxin but also for many other potential GB drugs is the inefficiency in passing the blood-brain-barrier and in targeting the GB tissue [27]. Recently a nanomedicine approach using a cancer cell–mitochondrial hybrid membrane including a polymer loaded with Gboxin that reacts with ROS called HM-NPs@G and ultimately improves blood-brain barrier penetrance as well as enables a specific release to tumor cells was reported [27]. Certainly, a parallel AMPK inhibition could represent a promising new approach for evaluation as GB treatment.

## Conclusion

S-Gboxin is effective in human glioma cells under the conditions of the tumor microenvironment. Starvation enhanced the effects of S-Gboxin with a faster and inefficient metabolism of glucose resulting in enhanced cell death and lower ability to migrate. The ISR, while getting activated by S-Gboxin, did not mediate therapy resistance. In contrast parallel AMPK inhibition enhanced S-Gboxin efficacy especially when AMPK was acutely inhibited by pharmacological inhibitors. A combinatory approach of S-Gboxin together with pharmacological AMPK inhibition could therefore be a promising GB-specific therapy approach which warrants further investigation e.g., in animal models.

## Material and methods

### Cell lines and cell culture

LN-229 were purchased from the American Type Culture Collection (ATCC). LNT-229 and LN-229 cells only differ in their p53 status and LNT-229 (TP53 wildtype) were a kind gift of N. de Tribolet (Lausanne, Switzerland) [12, 22]. LNT-229 cells were authenticated using STR analysis by Multiplexion (Heidelberg, Germany) and their STR profile matched with the profile of LN-229. G55T2 cells were a kind gift of Manfred Westphal and Kathrin Lamszus (Hamburg, Germany) [28]. Immortalized human astrocytes NHA-E6/E7/hTERT [29, 30] were purchased from the Department of Neurological Surgery of the University of California, San Francisco (San Francisco, CA, USA). All cell lines were cultured at 37 °C under a 5% CO_2_ atmosphere. For culturing, Dulbecco’s modified Eagle medium (DMEM) containing 10% fetal calf serum (FCS) (Thermo Fisher Scientific, Hamburg, Germany), 100 IU/ml penicillin and 100 μg/ml streptomycin (Life Technologies, Karlsruhe, Germany) was used. Cells were passaged at a confluence of approximately 90%. GL-261 cells were purchased from ATCC and cultivated in Dulbecco’s modified Eagle medium (DMEM) containing 10% fetal calf serum (FCS) (Thermo Fisher Scientific, Hamburg, Germany), 100 IU/ml penicillin and 100 μg/ml streptomycin (Life Technologies, Karlsruhe, Germany). Primary human astrocytes were a gift from Stefan Liebner (Frankfurt) and were cultured in human astrocyte growth medium (PELOBiotech, Planegg/Martinsried, Germany) containing 10% FCS. GB stem-like cells NCH644 and NCH690 were purchased from Cytion CLS (Eppelheim, Germany) and were cultured in Neurobasal A medium (Thermo Fisher Scientific, Dreieich, Germany) supplemented with 1 × B27 supplement (Thermo Fisher Scientific, Dreieich, Germany), 2 mM glutamine (Thermo Fisher Scientific, Dreieich, Germany), 1U/ml heparin, 100 IU/ml penicillin and 100 µg/ml streptomycin (Life Technologies, Karlsruhe, Germany) and 20 ng/ml EGF and FGF-2 (ReliaTech GmbH, Wolfenbüttel, Germany). Adherent GB stem-like cells were detached and separated using Accutase (Sigma-Aldrich/Merck, Darmstadt, Germany).

All cell lines were regularly checked for mycoplasma contamination throughout the study and only contamination-free cells were used for experiments.

BAY-974 and BAY-3827 were kindly provided by the DCP (Donated Chemical Probes) program [18]. Gboxin and S-Gboxin were purchased from Selleckchem (Houston, TX, USA). All reagents, if not specified elsewhere, were purchased from Sigma/Merck (Darmstadt, Germany).

### Genetic cell line models

LNT-229 cells with stable *ATF4* suppression have been previously described [14]. LNT-229 and G55T2 cells with a knockout of both catalytic (α1 and α2) AMPK subunits (double knockout, DKO) were generated by using the CRISPR/Cas9 system and have also been previously described [16]. The ATF4 5: 5’ATF4.GFP plasmid was a gift from David Ron (Addgene plasmid # 21852; http://n2t.net/addgene:21852; RRID: Addgene_21852) [31] and was used to generate ATF4-reporter cells by repeated sorting (Supplementary Fig. 1). Following transduction and stable integration, the cells were expanded under selection culture conditions using DMEM supplemented with 10% fetal bovine serum, 1% penicillin-streptomycin and 10 µg/ml blasticidin at 37 °C in a humidified 5% CO₂ atmosphere. Cells were stimulated with 1 μM thapsigargin for 4 h to induce endoplasmic reticulum stress and to activate the ISR pathway. Cells were FACS-sorted based on ATF4-GFP induction and subsequently cultured for recovery. Afterwards, cells were FACS-sorted again this time selecting for the absence of a GFP signal to ensure effective reporter turn-off in absence of ISR induction. This process was repeated several times until a functionally stable reporter population was established, exhibiting robust and reproducible GFP expression in response to ISR induction.

### Induction of hypoxia

For hypoxic conditions, anaerobic cell culture was achieved using GasPak pouches (Becton-Dickinson, Heidelberg, Germany). Serum-free DMEM containing 2 mM glucose was used for both hypoxic (0.1% O₂) and normoxic (21% O₂) experiments [32]. Glioma cells were seeded at a density of 120,000 cells per well in 24-well plates or 20,000 cells per well in 96-well plates and incubated overnight to facilitate attachment. Following a media change, cells were either exposed to hypoxia in GasPak pouches or left under normal atmosphere for the specified duration.

### Cell density and viability assay

Cell density was evaluated using crystal violet (CV) staining. A total of 20,000 cells were seeded per well in 96-well plates. After overnight incubation, the culture medium was replaced to match the experimental conditions, and CV staining was performed as previously described [12]. Cell viability was determined by quantifying propidium iodide (PI) uptake via flow cytometry following methods established in prior studies [14]. Data acquisition was conducted using a BD Canto II flow cytometer, and subsequent analysis was performed with BD FACS Diva software version 6.1.3.

### Immunoblot assay

Immunoblot analyses were performed following a standard protocol [33] utilizing the following antibodies: ATF4 (11815S, Cell Signaling, Danvers, MA, USA) and beta-actin (#sc-1616, Santa Cruz Biotechnology, Santa Cruz, CA, USA). Secondary peroxidase-coupled anti-rabbit and anti-goat antibodies were purchased from Jackson ImmunoResearch (#111-036-144; West Grove, PA, USA) and Santa Cruz Biotechnology (#sc-2020) respectively. Full length uncropped original western blots are provided in a separate supplemental material file (Supplementary file “Uncropped western blots”).

### Seahorse-based metabolic flux analysis

Respiration and extracellular acidification in different cell lines were evaluated using Seahorse XF96 extracellular flux analyzer (Agilent). Cells were plated in Seahorse 96-well cell culture plates at 3 × 10^4^ cells/well 1 day before the assay, pre-treated with 8 µM S-Gboxin for 5 h, and equilibrated in Seahorse DMEM medium (103575, Agilent) supplemented with 25 mM L-glucose and 2 mM L-glutamine prior to assay. Cells were treated with 2.5 µM oligomycin (Sigma-Aldrich), 1 µM carbonyl cyanide 3-chlorophenylhydrazone (CCCP, Sigma-Aldrich), 1 µg/ml antimycin (Sigma-Aldrich) and 2.5 µM rotenone (Sigma-Aldrich). OCR and extracellular acidification rates (ECAR) were monitored in real time after injection of each compound. Shown are representative OCR and ECAR traces of three independent experiments.

### Oxygen consumption assay

To measure the oxygen consumption rate, 120,000 cells were seeded in 24-well plates and incubated overnight to allow cell attachment. Following treatment as specified, the cells were overlaid with sterile paraffin oil. Oxygen consumption was quantified using a fluorescence-based assay (PreSens, Regensburg, Germany).

### Migration assay

800,000 cells were seeded in 6-well plates. The cells were grown to a confluence of 100% and a scratch was performed with a pipet tip. Pictures were taken with the CellCyte (Cytena, Germany, Freiburg) at 37 °C under a 5% CO_2_ atmosphere in an incubator.

### Lactate and glucose measurements

A total of 120,000 cells were seeded in 24-well plates. After overnight attachment, cells were treated according to the experimental design. Supernatants were collected after 10 h and centrifuged for storage at −80 °C. Analysis of glucose and lactate was performed using a Hitachi 917 biochemical analyzer, as described previously [24].

### Fluorescence microscopy

Fluorescence pictures were taken with the EVOS M5000 (Invitrogen/Thermo Fisher Scientific, Hamburg, Germany). 4× magnification was used in the transmission channel and the GFP channel.

### Statistical analysis

For evaluation of statistical significance GraphPad Prism 10 (version 10.2.23) software was used. For multiple comparison, one-way or two-way ANOVA was used, depending on if there was one or two variables. Tukey’s multiple comparison was used as a post-hoc test. Students *t*-test was used if only two mean values should be compared. Each experiment was performed with a sample size of 2–6 biological replicates (indicated with n at each figure), depending on the experiment type. The number of biological replicates was determined from the experience of previous experiments. Each experiment was repeated several times. A similar variance between the groups was estimated because the biological replicates were treated identically within each group. Thus, the variation within each group was expected to be small and similar between all compared groups. No data points were excluded in the experiments.

## Supplementary information

Supplementary figures

Uncropped western blots

## Data Availability

The datasets used or analyzed during the current study are available from the corresponding author on reasonable request.
