# Supplementary material for: Targeting glioblastoma mitochondrial metabolism with S-Gboxin induces cytotoxicity under conditions of the tumor microenvironment
Source: Cell Death Discov. 2026 Mar 27;12:181. doi: 10.1038/s41420-026-03072-4 (PMC13066480; doi:10.1038/s41420-026-03072-4)
Supplement: Supplementary file 1 — Supplementary figures [file 41420_2026_3072_MOESM1_ESM.docx]

**Supplementary information**

**Targeting glioblastoma mitochondrial metabolism with S-Gboxin induces cytotoxicity under conditions of the tumor microenvironment**

**Supplementary figures:**


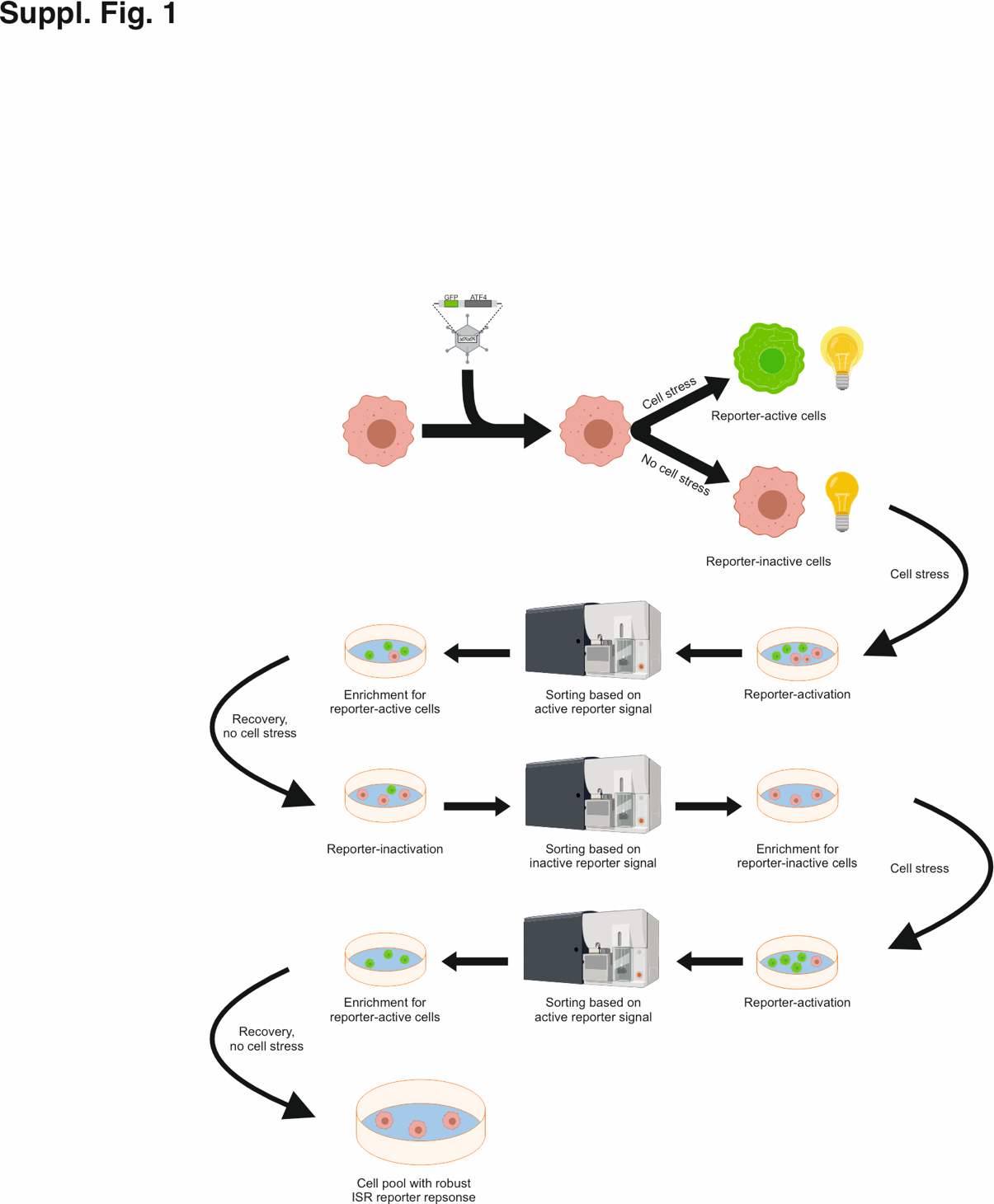


**Suppl. Fig. 1: Generation of LN-229 ATF4 reporter cell.**

Glioblastoma cell lines were transduced with the GFP-ATF4 fusion protein under the control of the endogenous ATF4 promoter. Thapsigargin was used to induce endoplasmic reticulum stress and activate the ISR. Cells were repeatedly FACS-sorted based on GFP signal as a measure of ISR induction and lack of a GFP signal when no ISR induction was present to ensure effective turn-off of the reporter. Figure created with biorender.com.


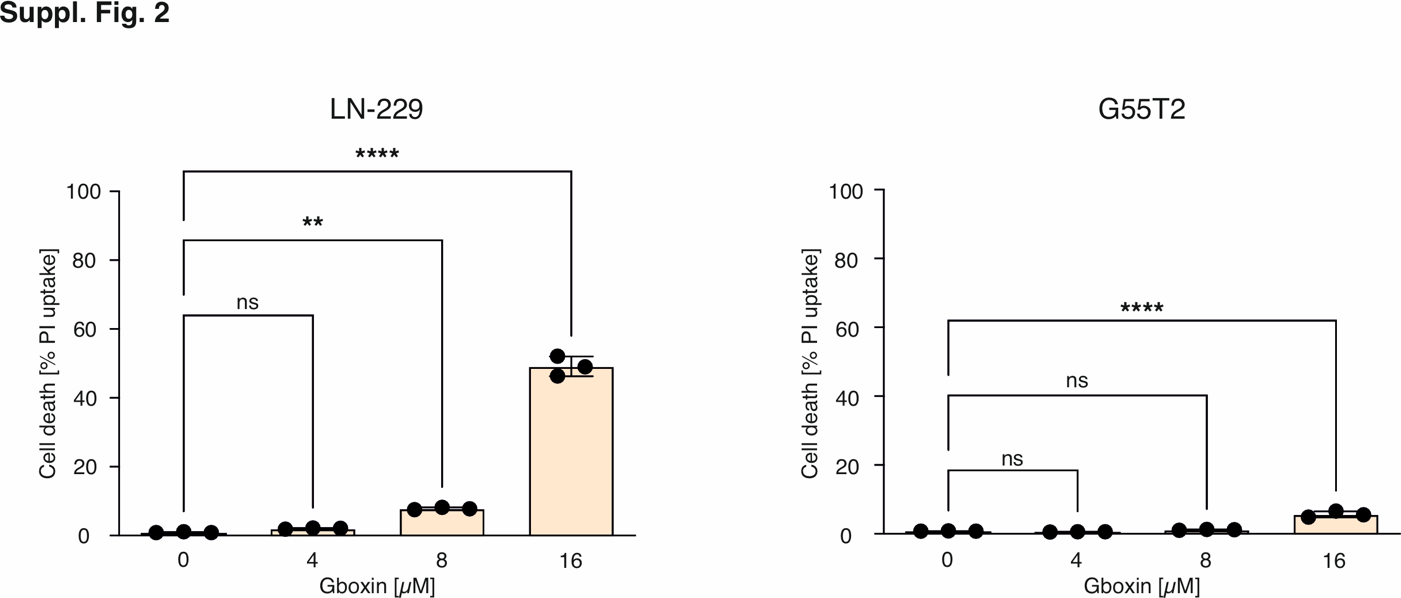


**Suppl. Fig. 2: Gboxin induces cell death in human glioma cell lines.**

Cell death of LN-229 (left panel) and G55T2 (right panel) cells was analyzed by PI-staining followed by FACS measurement after treatment with 4 µm, 8 µM and 16 µM Gboxin for 24 h in serum-free media. The equal amount of DMSO compared to 16 µM Gboxin was used as control (n=3, Mean and S.D. are presented. ns = not significant, p-value *<0.05, **<0.01, ***<0.001, ****<0.0001; one-way ANOVA with Tukey`s multiple comparison).


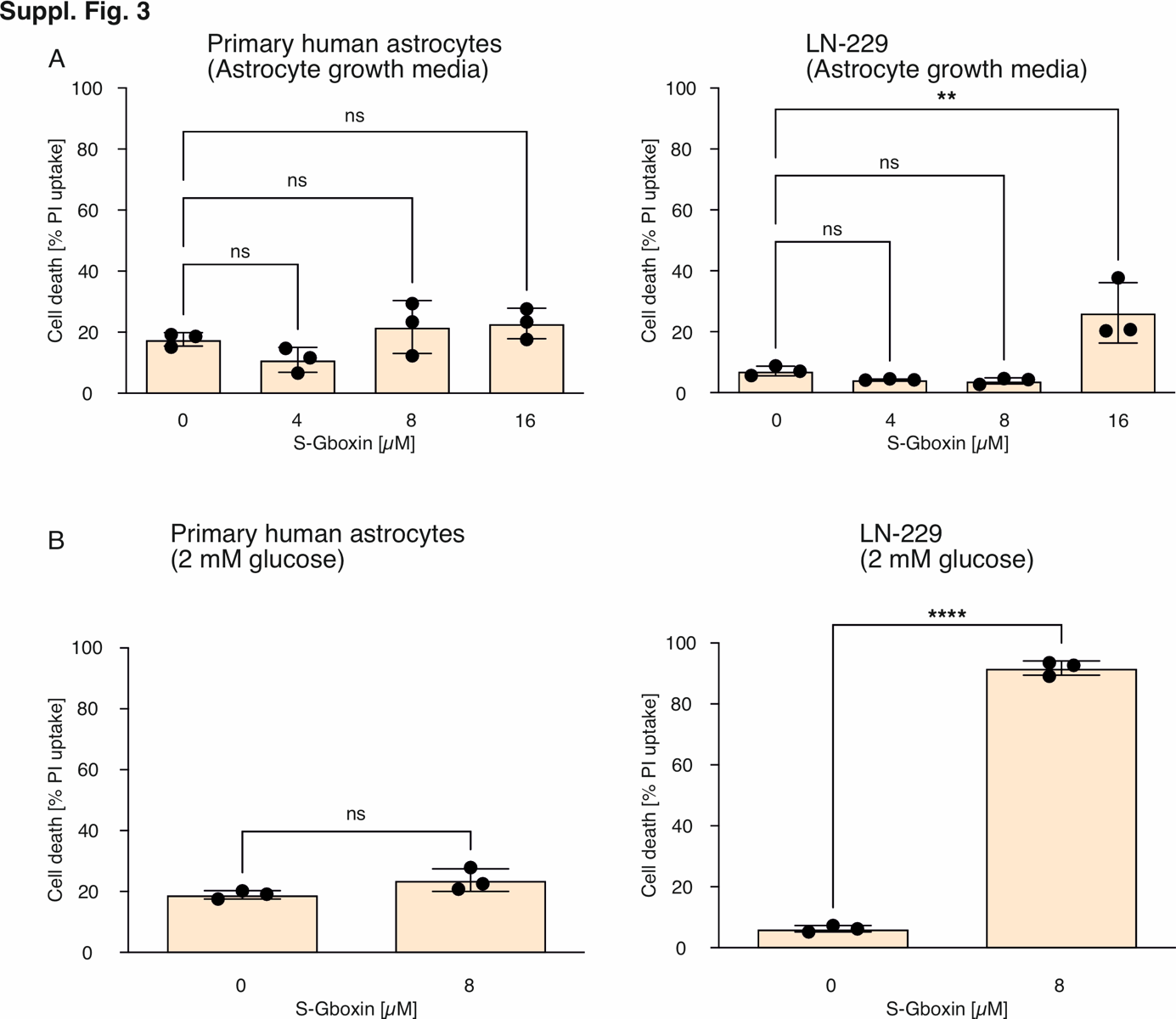


**Suppl. Fig. 3: S-Gboxin induces cell death under different culture conditions in LN-229 glioblastoma cell line while treatment in primary human astrocytes shows no effect.**

**A** Cell death of primary human astrocytes (left panel) and LN-229 cells (right panel) was analyzed under 4 µm, 8 µM and 16 µM S-Gboxin for 24 h in human astrocyte growth media. The equal amount of DMSO compared to 16 µM S-Gboxin was used as control (n=3, Mean and S.D. are presented. ns = not significant, p-value *<0.05, **<0.01, ***<0.001, ****<0.0001; one-way ANOVA with Tukey`s multiple comparison).

**B** The same experiment was repeated in DMEM containing 2 mM glucose with or without 8 µm S-Gboxin. The equal amount of DMSO compared to 8 µM S-Gboxin was used as control. Cell death was analyzed by PI-staining and FACS analysis (n=3, Mean and S.D. are presented. ns = not significant, p-value *<0.05, **<0.01, ***<0.001, ****<0.0001; students t-test).

**
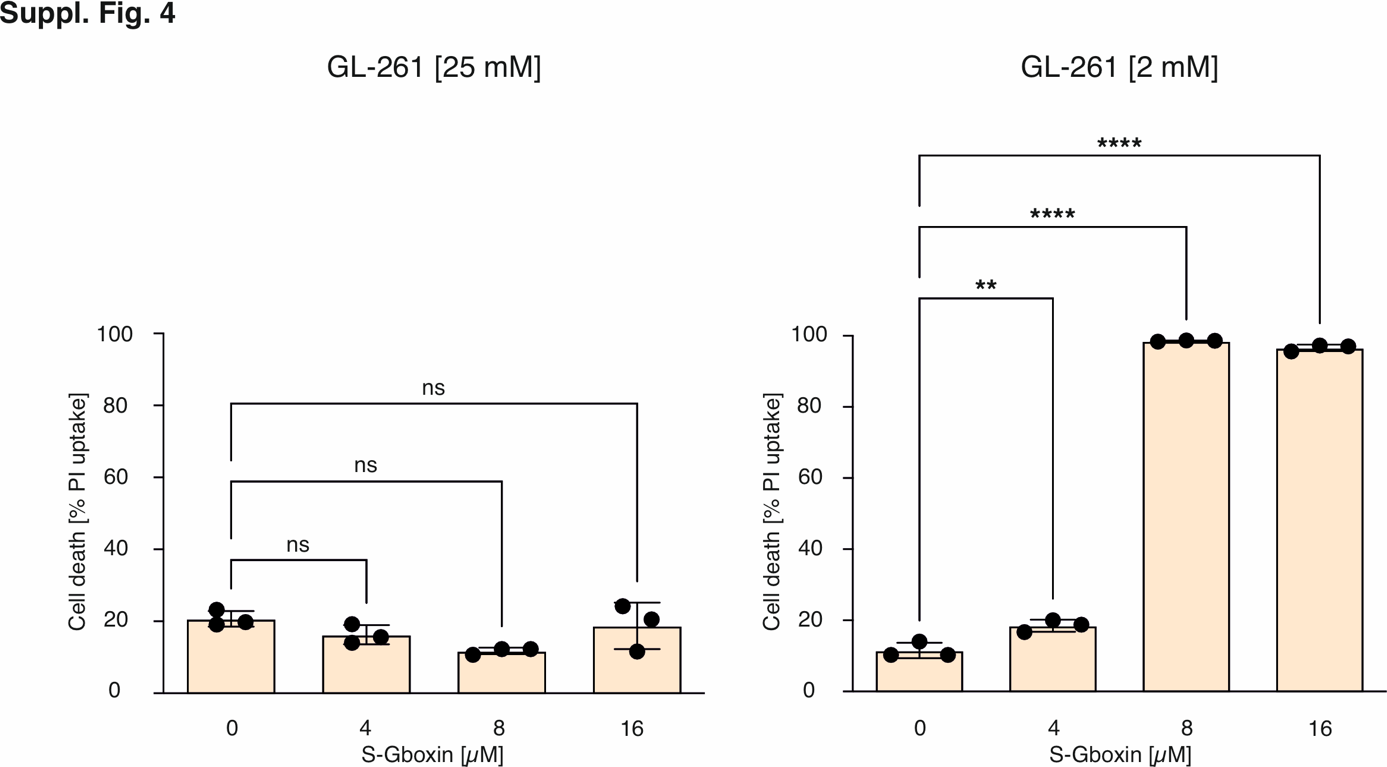
**

**Suppl. Fig. 4: S-Gboxin induces cell death in the mouse glioma cell line GL-261 under nutrient deprivation.**

Mouse glioma cell line GL-261 was treated with 4 µM, 8 µM or 16 µm S-Gboxin in serum-free media (left panel) or DMEM containing 2 mM glucose (right panel). Equal amount of DMSO compared to the highest S-Gboxin concentration was used as control. Cell death was analyzed by PI-staining and FACS after 24 h incubation (n=3, Mean and S.D. are presented. ns = not significant, p-value *<0.05, **<0.01, ***<0.001, ****<0.0001; one-way ANOVA with Tukey`s multiple comparison).


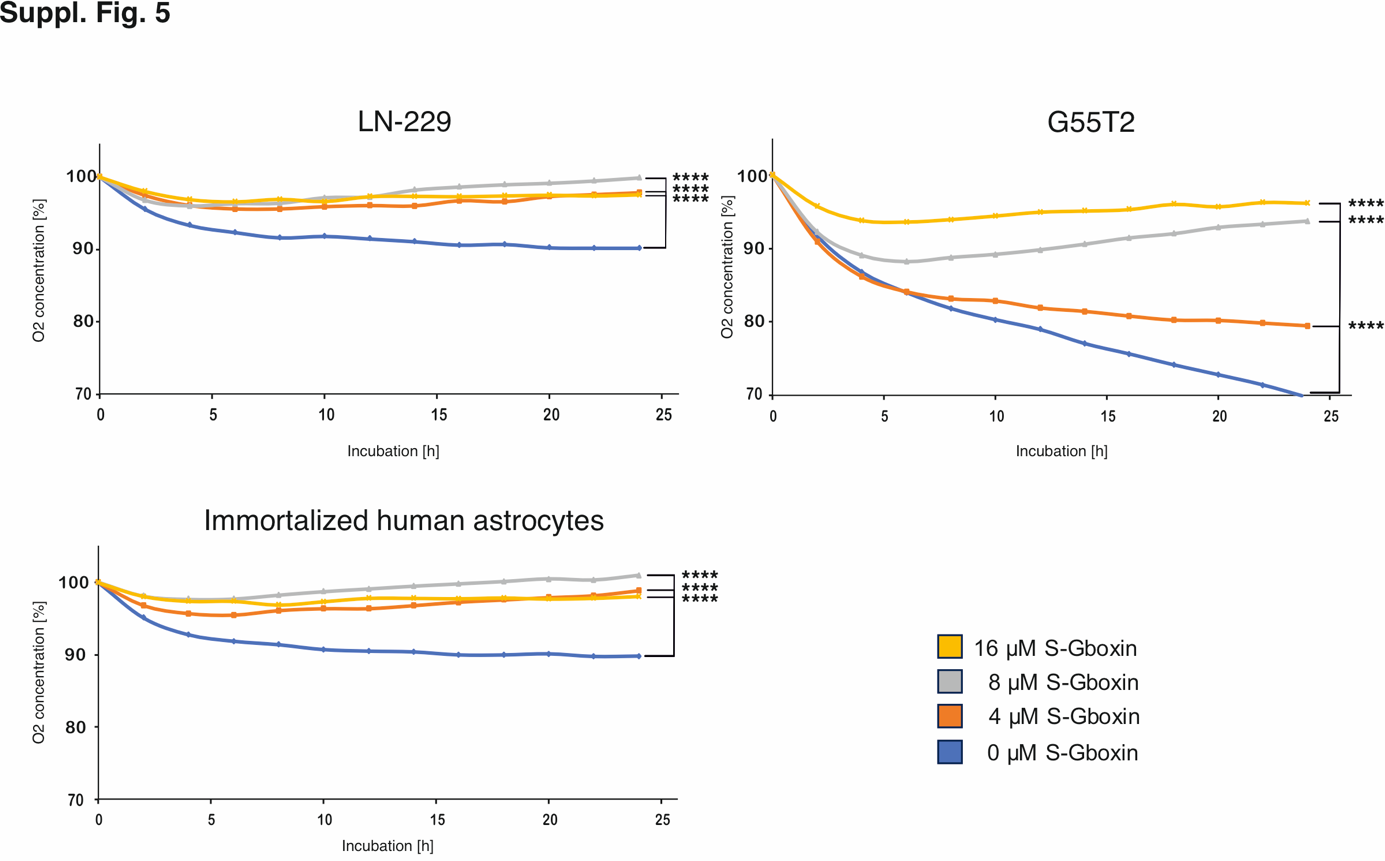


**Suppl. Fig. 5: Oxygen consumption due to S-Gboxin treatment.**

The O_2_ consumption of LN-229 (upper left panel), G55T2 (upper right panel) and immortalized human astrocytes (lower left panel) was determined for 24 h in serum-free media with treatment of 4 µM (orange), 8 µM (gray) and 16 µM (yellow) S-Gboxin. Statistical significance was calculated tested by endpoint analysis (n=3, Mean is presented. p-value *<0.05, **<0.01, ***<0.001, ****<0.0001; one-way ANOVA with Tukey`s multiple comparison).


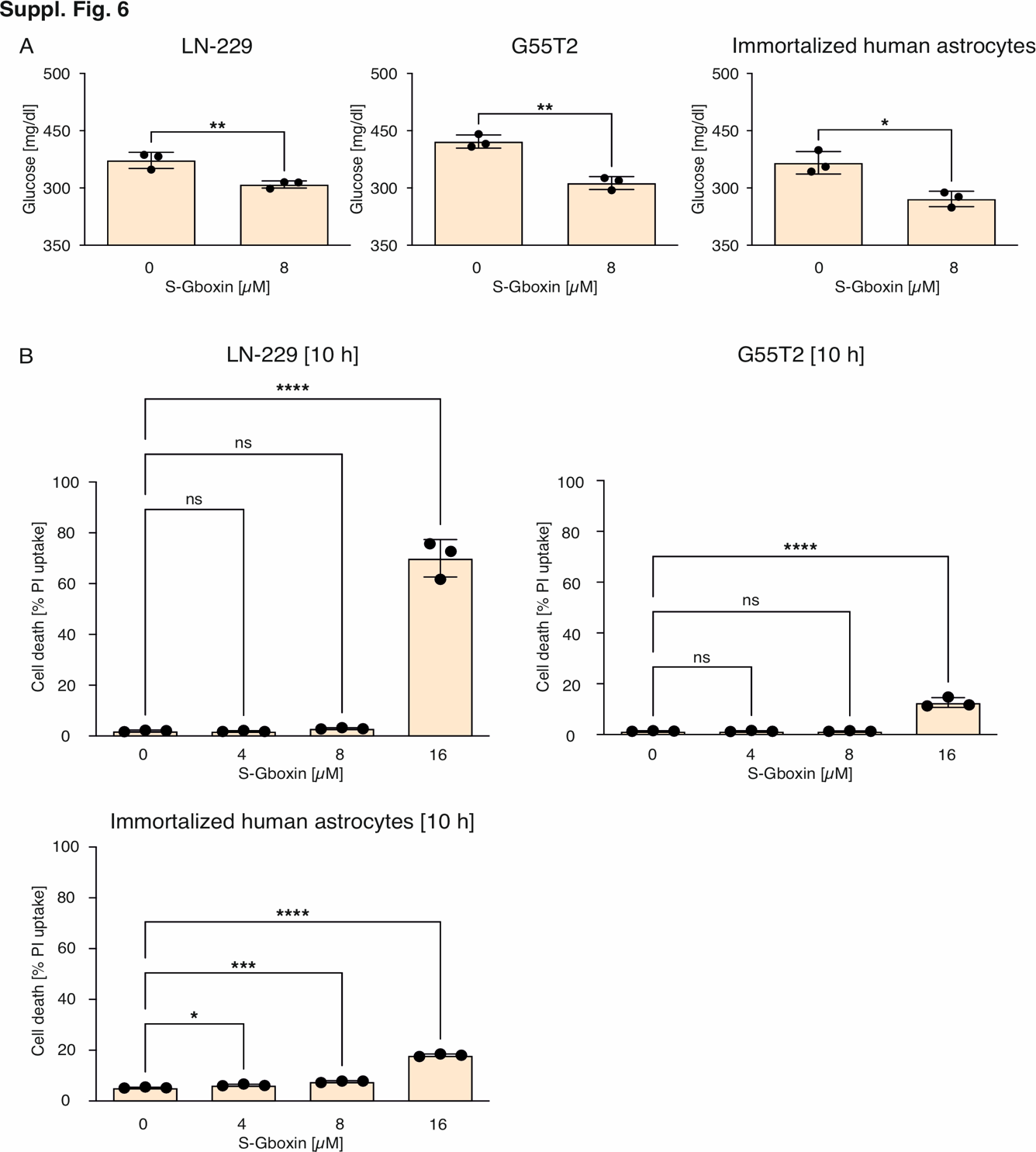


**Suppl. Fig. 6: Glucose consumption is enhanced under S-Gboxin treatment.**

**A** Human glioma cell lines LN-229 (left panel) and G55T2 (middle panel) as well as immortalized human astrocytes (right panel) were incubated for 10 h with 8 µm S-Gboxin. Equal amount of DMSO was used as control. Afterwards, glucose concentration in mg/dl was determined (n=3, Mean and S.D. are presented. p-value *<0.05, **<0.01, ***<0.001, ****<0.0001; students t-test).

**B** The cell death after 10 h treatment with 4 µM, 8 µM and 16 µM S-Gboxin in these cell lines was determined using PI-staining and FACS. The equal amount of DMSO compared to 16 µM S-Gboxin was used as control (n=3, Mean and S.D. are presented. ns = not significant, p-value *<0.05, **<0.01, ***<0.001, ****<0.0001; one-way ANOVA with Tukey`s multiple comparison).

**
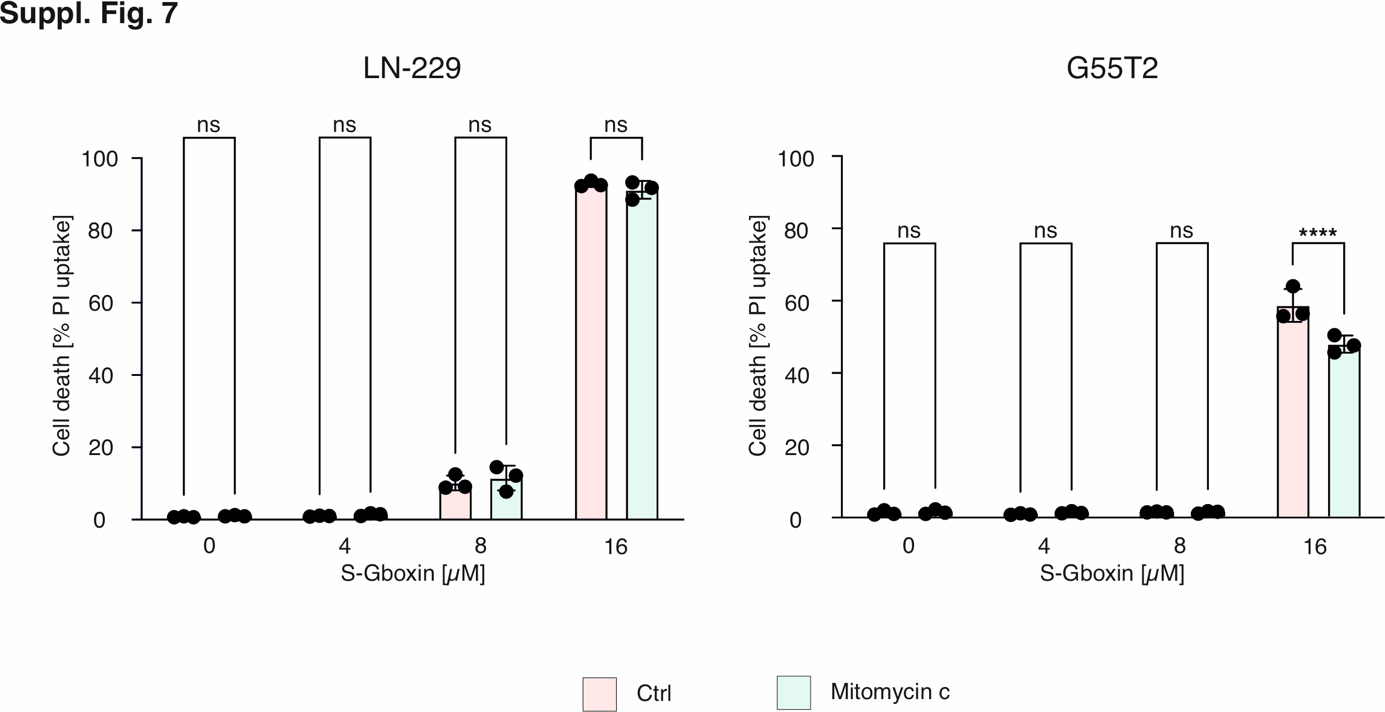
**

**Suppl. Fig. 7: Mitomycin c does not affect S-Gboxin efficacy under 4 µM and 8 µM S-Gboxin.**

Human glioma cell lines LN-229 (left panel) and G55T2 (right panel) were incubated for 2 h with 20 µg Mitomycin c (green) or just incubated in serum-free media (red). Afterwards, cells were treated with 4 µM, 8 µM or 16 µM S-Gboxin in serum-free media for 24 h. Equal amount of DMSO was used as control (n=3, Mean and S.D. are presented. p-value *<0.05, **<0.01, ***<0.001, ****<0.0001; two-way ANOVA with Tukey`s multiple comparison).


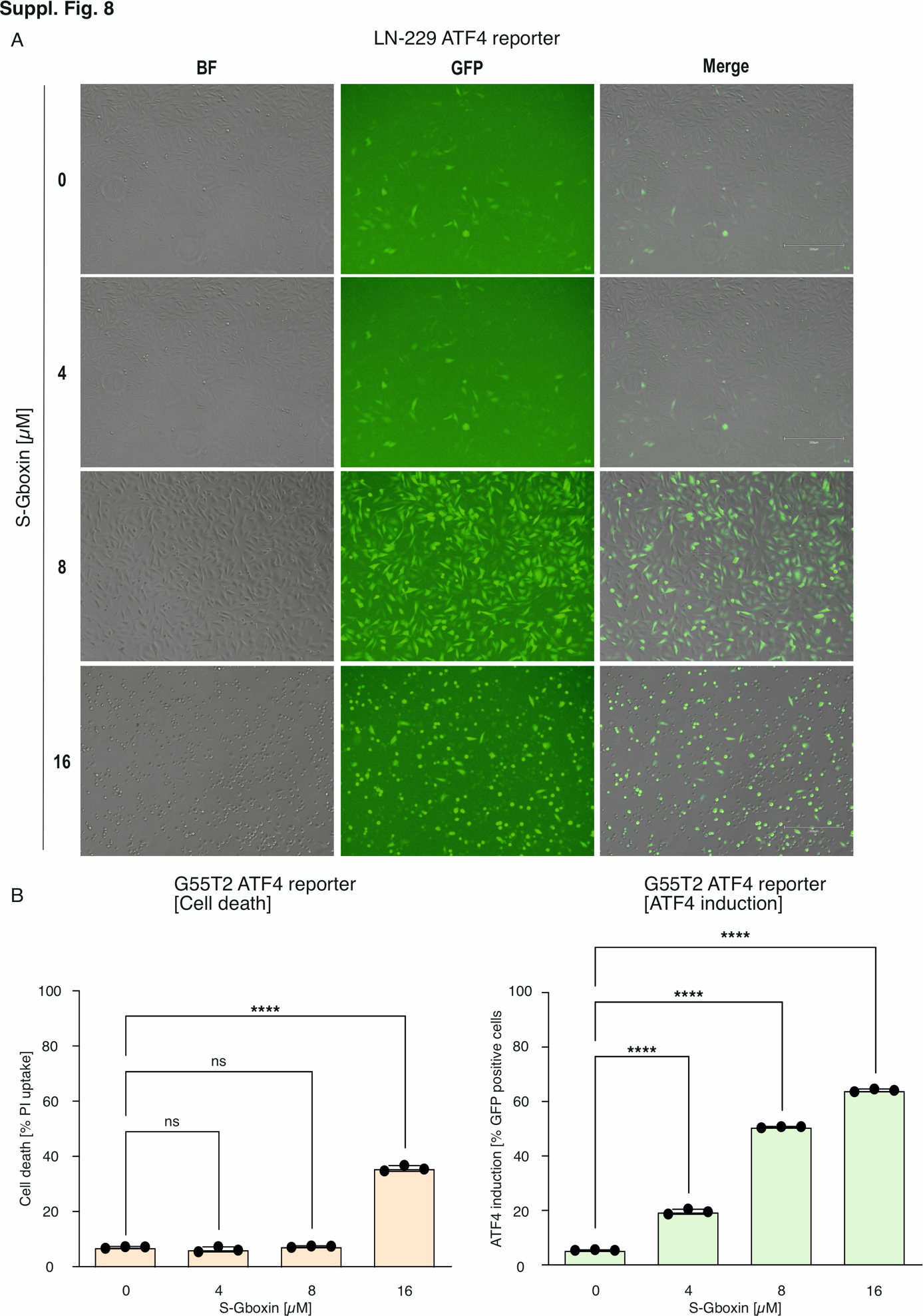


**Suppl. Fig. 8: S-Gboxin activates ATF4 in G55T2 and LN-229 cells.**

**A** LN-229 ATF4 reporter cells were treated with 4 µM, 8 µM or 16 µM S-Gboxin or a DMSO control. Representative pictures were taken after 24 h. Bright field (left) and GFP signal (middle) were recorded and merged (right). **B** Cell death (left) and GFP signal (right) were measured after 24 h in G55T2 ATF4 reporter cells. Cell death was quantified using PI-stain followed by FACS. The cells were treated with 4 µM, 8 µM and 16 µM S-Gboxin in serum-free media (n=3, Mean and S.D. are presented. ns = not significant, p-value *<0.05, **<0.01, ***<0.001, ****<0.0001; one-way ANOVA with Tukey`s multiple comparison).


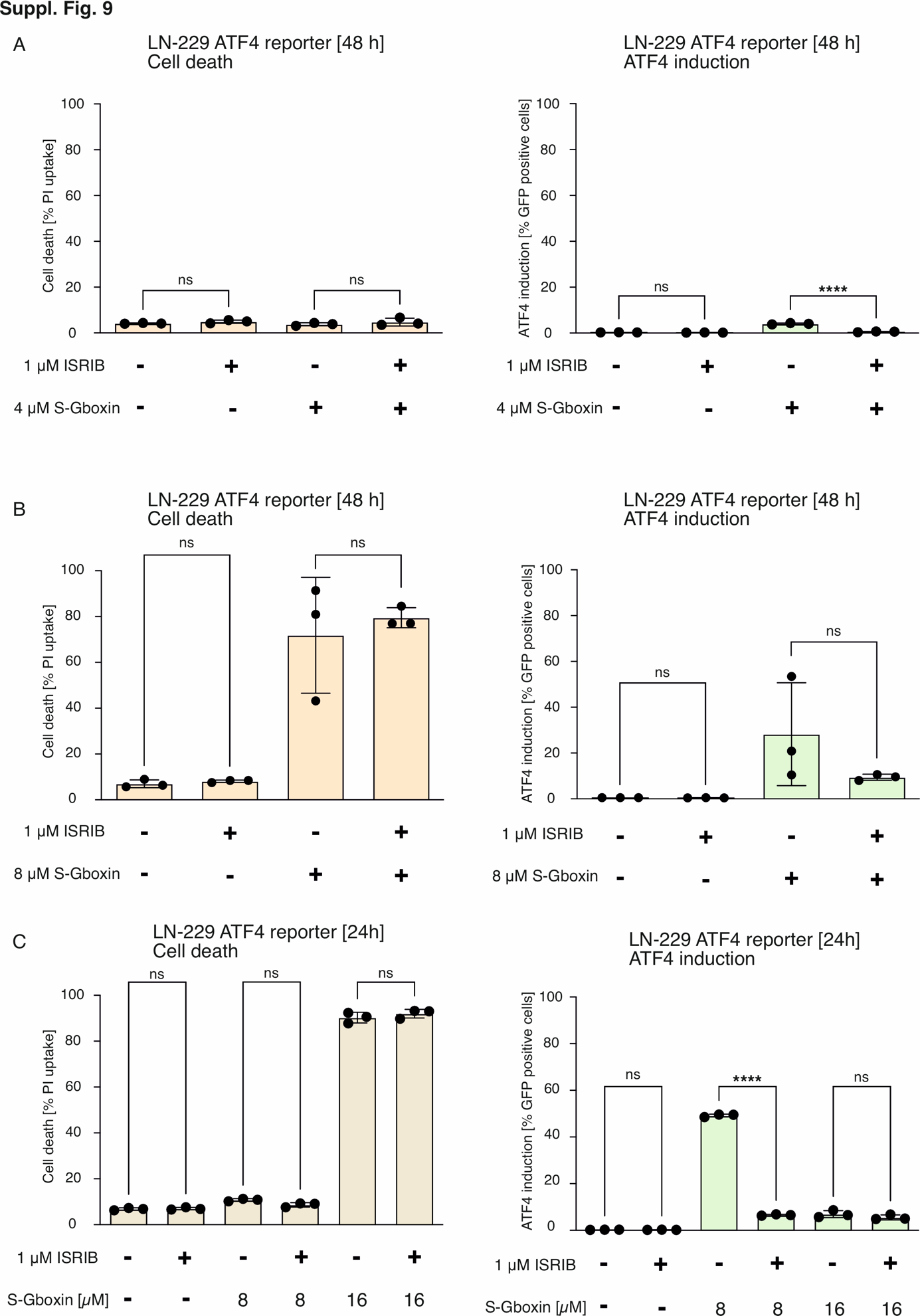


**Suppl. Fig. 9: Pharmacological ATF4 inhibition does not enhance S-Gboxin-dependent cell death.**

LN-229 ATF4 reporter cells were treated with S-Gboxin, 1 µm ISRIB or S-Gboxin and ISRIB together. **A** 4 µm S-Gboxin were used for 48 h in SFM. Cell death (left panel) was quantified after PI-staining and GFP signal (right panel) was measured (n=3, Mean and S.D. are presented. ns = not significant, p-value *<0.05, **<0.01, ***<0.001, ****<0.0001; one-way ANOVA with Tukey`s multiple comparison).

**B** The same experiment was performed using 8 µM S-Gboxin. (n=3, Mean and S.D. are presented. ns = not significant, p-value *<0.05, **<0.01, ***<0.001, ****<0.0001; one-way ANOVA with Tukey`s multiple comparison)

**C** Also, 8 µM and 16 µM S-Gboxin were used for 24 h (n=3, Mean and S.D. are presented. ns = not significant, p-value *<0.05, **<0.01, ***<0.001, ****<0.0001; one-way ANOVA with Tukey`s multiple comparison).


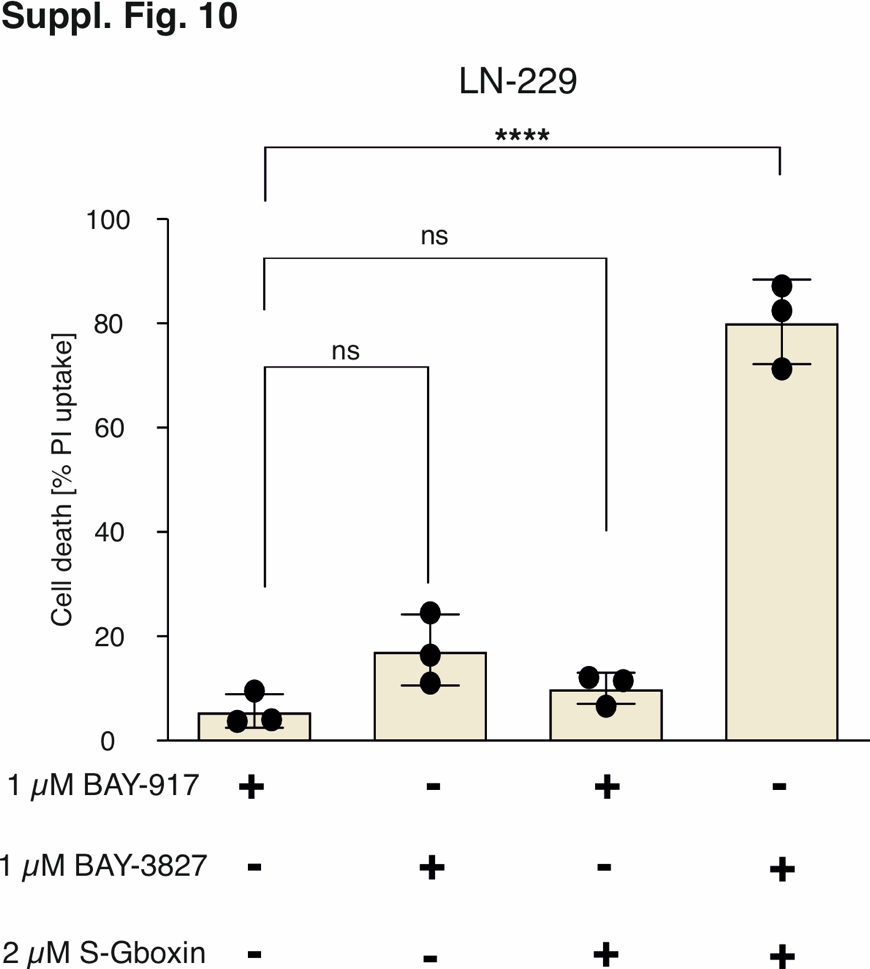


**Suppl. Fig. 10: AMPK inhibition in combination with S-Gboxin treatment leads to enhanced cell death in LN-229 cells.**

LN-229 cells were treated for 24 h with 2 µM S-Gboxin and/or with 1 µm AMPK-inhibitor BAY-3827 or the control substance BAY-917 in DMEM containing 2 mM glucose. Cell death was quantified using PI and FACS analysis (n=3, Mean and S.D. are presented. ns = not significant, p-value *<0.05, **<0.01, ***<0.001, ****<0.0001; one-way ANOVA with Tukey`s multiple comparison).


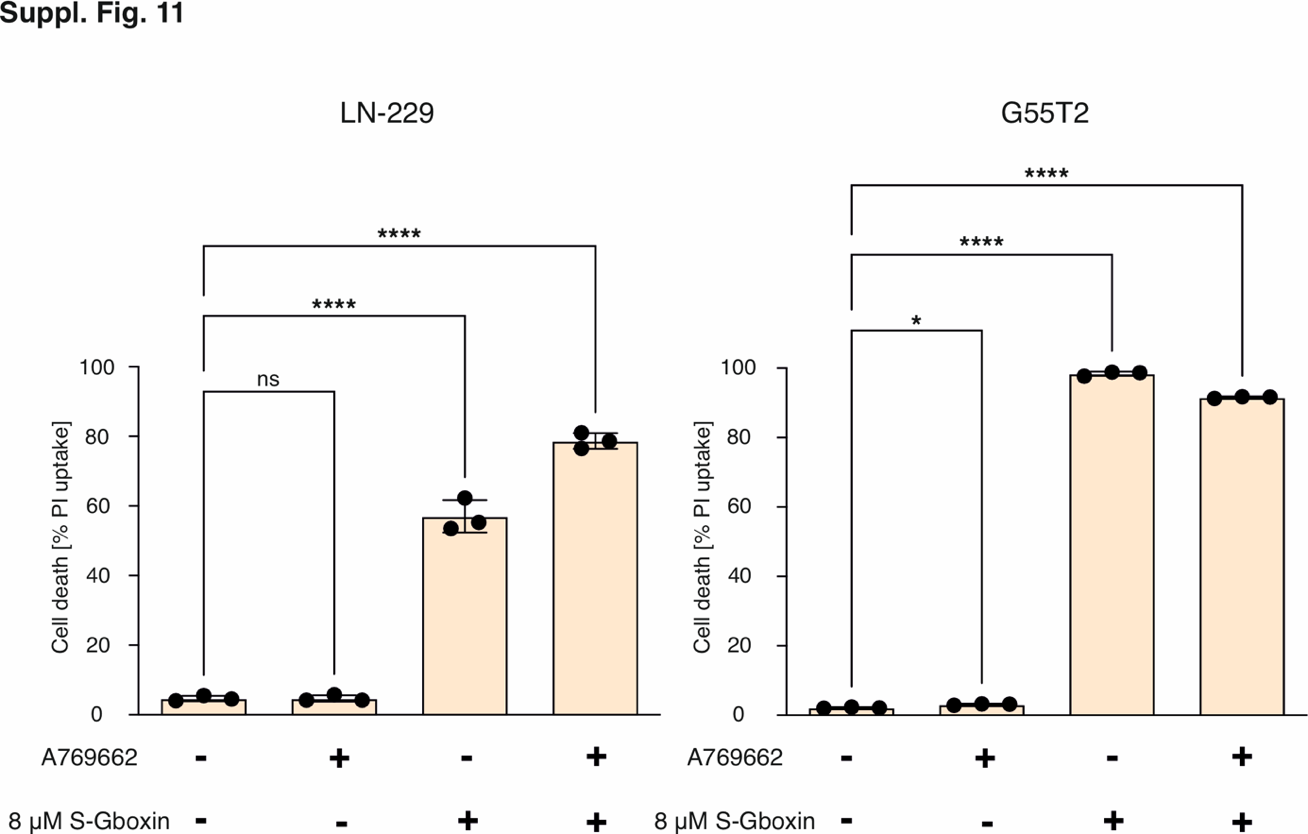


**Suppl. Fig. 11: Activation of AMPK does not protect from S-Gboxin.**

LN-229 cells (left panel) and G55T2 cells (right panel) were treated with 100 µm A769662, 8 µM S-Gboxin or A769662 together with S-Gboxin in DMEM containing 2 mM glucose. DMSO was used as control. Cell death was measured using PI-staining and FACS after 24 h (n=3, Mean and S.D. are presented. ns = not significant, p-value *<0.05, **<0.01, ***<0.001, ****<0.0001; one-way ANOVA with Tukey`s multiple comparison).
