# Supplementary material for: Targeting glioblastoma mitochondrial metabolism with S-Gboxin induces cytotoxicity under conditions of the tumor microenvironment
Source: Cell Death Discov. 2026 Mar 27;12:181. doi: 10.1038/s41420-026-03072-4 (PMC13066480; doi:10.1038/s41420-026-03072-4)
Supplement: Supplementary file 2 — Uncropped western blots [file 41420_2026_3072_MOESM2_ESM.docx]

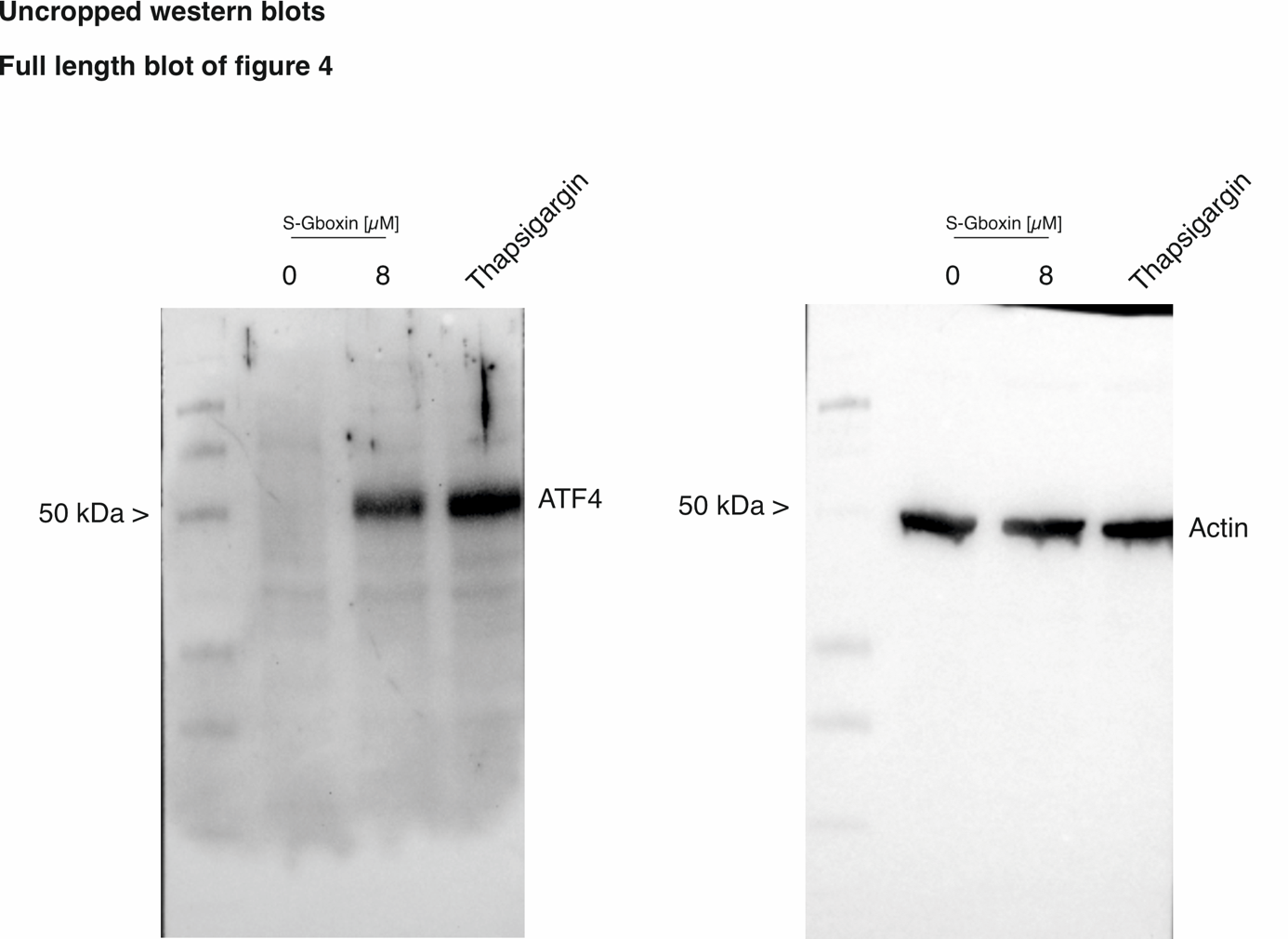


**Uncropped western blot of figure 4.**

Full-length immunoblot of figure 4. ATF4 and actin signal after 24 h incubation of LN-229 cells in serum-free media with 0 µM S-Gboxin, 8 µM S-Gboxin or 1 µM thapsigargin.
